# Supplementary material for: Construct validation and measurement invariance of the Parasocial Relationships in Social Media survey
Source: PLoS One. 2024 Mar 28;19(3):e0300356. doi: 10.1371/journal.pone.0300356 (PMC10977705; doi:10.1371/journal.pone.0300356)
Supplement: Attachment — Submitted filename: PLOS One Review Letter.docx [file pone.0300356.s001.docx]

PONE-D-23-12810
Construct validation and measurement invariance of the Parasocial Relationships in Social Media Survey

Dear Dr. Sudzina,

We would like to thank you and the reviewers for your insightful comments on our manuscript and for the opportunity to improve our manuscript. We appreciate the reviewers’ acknowledgment of the merits of the study and are especially grateful for the important questions raised and areas of improvement identified in the comments. As a result, we have incorporated the reviewers’ recommendations in our revised manuscript. To ease the review process, we have listed the reviewers’ comments and a corresponding narrative describing our response. We look forward to your thoughts on our revisions. We hope you agree that the manuscript is now in a better position to be informative and valuable to the *PLOS One* readership.

Sincerely,

The authors

| Reviewer | Comment | Response/Revision |
| --- | --- | --- |
| 1 | My major critique of this paper concerns the elements of the scale itself, in that they are not indicative of a relationship so much as an attitude of interest toward a celebrity. Only the items in the “Interaction With” scale (ironically, given the discussion in the literature regarding the difference between parasocial interactions and relationships), tap into the socioemotional basis of a relationship, and they are limited to friendship. This measure does not seem to allow for participants whose relationship with a media figure is romantic or that of a mentor/mentee (or anything other than friendship). In that sense, the notion that this survey measures PSR per se is as flawed as the measures (PSI, API) from which items were derived. If these relationships can “feel as real to viewers as real-life relationships” (p. 3) then why aren’t any of the items focused on identifying the social and emotional affordances of real relationships that are available in parasocial relationships? This scale appears to me not to measure an imagined relationship so much as a set of behaviors and attitudes connected to a media figure. | Thank you for your insights and the opportunity to clarify our approach.  Regarding the “Interaction With” scale, we believe its inclusion is not ironic but rather integral to the concept of parasocial relationships. As noted in the literature (e.g., Vorderer, 1996; Dibble et al., 2016; Boyd et al., 2022), parasocial relationships are formed and strengthened through repeated parasocial interactions. This is discussed in detail on page 7, paragraph 1 of the Parasocial Relationships section of our paper.  The argument that the scale not accommodating to participants who perceive their relationship with a media figure as romantic or that of a mentor/mentee is correct. However, as both romantic (Collins et al., 2009) and mentor/mentee relationships (Hill et al., 2022) necessitate reciprocation, they fall outside the scope of parasocial relationships, which by definition (Horton and Wohl, 1956) cannot be reciprocated by the celebrity. This is elaborated on page 6, paragraph 1 of the Parasocial Relationships section.  We would also like to highlight that our survey does include items addressing the social and emotional affordances of real relationships. Affordances, as defined by Gibson (1979), are what the environment offers, provides, or furnishes, either for good or ill. Social and emotional affordances refer to possibilities for social interactions (de Carvalho, 2020) and ways to transmit or collect emotional meaning (Vallverdú & Trovato, 2016), respectively. Several items including “I would follow [NAME] on another account if they create one on the same social media site that I already follow them on.”, “[NAME] keeps me company when viewing their content.”, and “[NAME] makes me feel as if I am with a friend.” deal with possibilities for social interactions while others such as “I hope [NAME] achieves their goals.”, “I care about what happens to [NAME].”, and “[NAME] understands the kinds of content I want to see.” deal with emotional aspects.  Lastly, we agree that a relationship is an abstract concept and cannot be measured directly. Therefore, we have chosen to measure behaviors and attitudes connected to the media figure as indicators of the relationship construct. We believe this approach provides a practical and effective way to study parasocial relationships. |
| 1 | The method was a little odd in not starting with a description of the sample and in focusing on describing the analyses conducted, which I think of as information that typically appears in Results. Perhaps such a format is standard for measurement papers. | The method section has been reordered to present the description of the sample and recruitment methods before describing the analysis that has been conducted. |
| 1 | In the Results section, I was a little confused as to why a lack of correlation between the PRISM subscales and the year in which the participant first started following the celebrity was considered evidence of discriminant validity. Relationship models (for real relationships) emphasize not just frequency of interactions, but the duration of the relationship. Why wouldn’t the same be true for parasocial relationships? | While it is true that the duration of a relationship can be important in traditional interpersonal relationships, parasocial relationships are fundamentally different in nature. Parasocial relationships are fundamentally one-sided, where an individual develops a strong sense of connection, intimacy, or familiarity with someone they don’t know, most often celebrities or media personalities. These relationships exist only in the mind of the individual, who experiences a bond despite the lack of reciprocity.  We have included additional information in both the results and discussion to address this. |
| 1 | Was “interact with” defined for participants? I’m not sure if it includes simply reading/watching the media figure’s posts or if, at minimum, the participant had to respond to the post in some way (like it) for that to count as an interaction—or perhaps it was left open to interpretation by the participant? | While one of the constructs in the survey is named “Interaction with”, the survey itself does not ask participants about how they “interact with” the media figure nor does it state the name of the constructs being measured, so it was not defined there. In the demographic questions, participants were asked “On average, how often do you interact with [NAME]’s posts online?” for analysis of the convergent validity. This was defined for the participants as “engaging with or viewing content that they [the celebrity] has been a part of”. This has been added to paragraph 3 of the results section. |
| 1 | I might not fully understand the section on measurement invariance but if the sample was separated by platform used (YouTube v. Twitch), how would that work with so few people reporting that they followed their media figures on Twitch? Isn’t this comparison rather unbalanced? | While the groups used in the measurement invariance analysis are unbalanced, a substantial number of respondents (n = 108) primarily followed the celebrity on Twitch. However, it’s important to note that large imbalances in group size might affect the results of factorial invariance studies since many aspects of CFA are influenced by sample size (e.g., Chi-square, standard errors). To bolster our findings, we implemented an approach suggested by Yoon & Lai (2018) in which random samples from the larger group (i.e., YouTube) were taken and measurement invariance was assessed. Conclusions for measurement invariance were confirmed in each of these analyses. We have added these findings to the manuscript. |
| 1 | Small things:  The second paragraph of the introduction in the section on Parasocial Relationships is enormous and covers multiple ideas. I suggest that it be split up. The end of this paragraph also states that the PRISM survey does not need to be modified for use in social media contexts, but it raised the question for me—why create a scale for use with social media rather than one that could be used for any context? Is that not possible? Does each context thus need its own survey instrument?  In Results, descriptions of the information presented in the tables should be adjacent to the tables themselves (e.g., discussion of measures of internal consistency, presented in table 2, came two pages later after table 4).  I noted a few typos throughout the manuscript. | Thank you for these suggestions. We have corrected typos throughout and rearranged the Results section to ensure the Tables better align with the text narrative.  We have also revised the introduction to better articulate the need for the PRISM survey and the unique role of social media in developing parasocial relationships. |
| 2 | 1 .Currently, there are some established PSI scales that have been widely adopted with high levels of reliability and validity, and some of them are developed within social media context, e.g. Dibble, J. L., Hartmann, T., & Rosaen, S. F. (2016). Parasocial interaction and parasocial relationship: Conceptual clarification and a critical assessment of measures . Regrettably, the introductory section lacks a clear delineation of the distinctive aspects of the authors' study and fails to elucidate the necessity for the re-creation of the scale. | There seems to be a misunderstanding surrounding the Dibble et al. (2016) article. The purpose of the article was to determine if one of the most widely cited scales (the PSI-Scale) and the newly created EPSI scale were in fact measuring parasocial interactions, not parasocial relationships. The article does not suggest there are any scales developed within a social media context, for parasocial interactions or relationships, nor any with high levels of reliability and validity.  The authors specifically caution against the use of existing measures in the literature due to the lack of sufficient research surrounding their validity. This caution is, in fact, the modification behind our current study. The PRISM survey, which has already been published (Boyd et al., 2022), is not being recreated in this study. Instead, we are conducting further construct validation and measurement invariance testing on the published survey. |
| 2 | 2. While the literature review has touched upon differences between social media and traditional media, such as interactivity, the justification for the necessity of revising the scale remains insufficient. Further clarification is required regarding the theoretical significance and practical application value of this scale revision. | We have revised the introduction, literature review, and discussion sections to include additional information regarding the necessity and practical application of the scale. Our study does not propose any revisions to the PRISM scale. Instead, it is a validation study aimed at reporting on the psychometric properties of the published scale. |
| 2 | 3. The demographic data for the sample should be provided in detail, outlining key demographic characteristics. Additionally, clear criteria for the selection of the 227 celebrities need to be explicitly stated. | Detailed demographic information is included in the paper. Originally at the end of the Methods section, it is now the third subsection in the Methods listed as “Participants in Current Study”. This includes information on the age, gender identification, ethnic breakdown, education levels, and employment status of respondents.  The 227 celebrities were identified by the participants themselves, not the researchers. This has been made more clear in paragraph 2 of the “Participants in Current Study” section. |
| 2 | 4. The correlation of the Identification With dimension is notably low. Therefore, the need for revision of this dimension is a matter that requires further consideration in the data analysis. | Thank you for your feedback on the “Identification With” dimension. We have revised the discussion section of the paper to address this concern and explain the possible reasons for the low correlation of this dimension with the frequency of interaction with the celebrity’s posts online. |
| 2 | 5. The discussion section needs a more comprehensive elucidation of the theoretical significance and practical value of this study. Furthermore, limitations of the research and future prospects are currently lacking in sufficient detail. | Thank you for this feedback. We have revised the discussion section to include more information on the theoretical significance and practical value of the study. We have also included limitations and directions for future research. |

Collins, W. A., Welsh, D. P., & Furman, W. (2009). Adolescent romantic relationships. *Annual Review of Psychology*, *60*(1), 631–652. https://doi.org/10.1146/annurev.psych.60.110707.163459

de Carvalho, E. M. (2020). Social Affordance. *Encyclopedia of Animal Cognition and Behavior*, 1–4. https://doi.org/10.1007/978-3-319-47829-6_1870-1

Gibson, J. J. (1979). The Theory of Affordances. In *The Ecological Approach to Visual Perception* (pp. 127–137). Lawrence Erlbaum Associates, Inc.

Hill, S. E., Ward, W. L., Seay, A., & Buzenski, J. (2022). The nature and evolution of the mentoring relationship in academic health centers. *Journal of Clinical Psychology in Medical Settings*, *29*(3), 557–569. https://doi.org/10.1007/s10880-022-09893-6

Vallverdú, J., & Trovato, G. (2016). Emotional affordances for Human–Robot Interaction. *Adaptive Behavior*, *24*(5), 320–334. <https://doi.org/10.1177/1059712316668238>

Myeongsun Yoon & Mark H. C. Lai (2018) Testing Factorial Invariance With Unbalanced Samples, Structural Equation Modeling: A Multidisciplinary Journal, 25:2, 201-213, DOI: 10.1080/10705511.2017.1387859

Yoon, M. & Lai, M. H. C. (2018). Testing factorial invariance with unbalanced samples. Structural Equation Modeling: A multidisciplinary journal. https://doi.org/10.1080/10705511.2017.1387859
